# Measurement of anisotropic volumetric resistivity in lithium ion electrodes†

**DOI:** 10.1039/d3ra06412c

**Published:** 2023-11-14

**Authors:** M. J. Lain, G. Apachitei, D.-E. Dogaru, W. D. Widanage, J. Marco, M. Copley

**Affiliations:** a WMG, University of Warwick Coventry CV4 7AL UK m.j.lain@warwick.ac.uk

## Abstract

Measurements of the electronic conductivity of lithium ion coatings are an important part of electrode development, particularly for thicker electrodes and in high power applications. A resistance measurement system with 46 probes has been used to characterise lithium ion electrodes, with different formulations and coat weights. The results show that the total through plane resistance is dominated by the interface resistance between the coating and the metal foil, rather than the volumetric resistivity of the coating. For coatings containing carbon nano-tubes, the in plane resistivities in the coating and perpendicular directions are different. A finite volume model was developed to help analyse and interpret the resistivity data.

## Abstract 2

Lithium ion electrodes with carbon fibres can show *X*–*Y* anisotropy in their electronic conductivity. The interface resistance between coatings and foils is much larger than the coating resistance; for cathodes the former is reduced by calendering.

## Graphical abstract

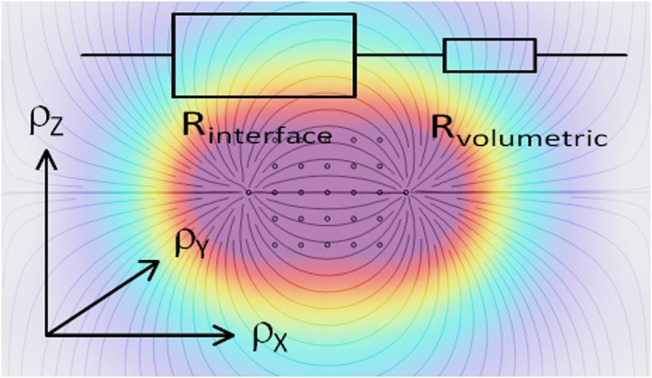

## Introduction

The manufacture of lithium ion cells involves a sequence of processing steps, each of which must be performed well to produce a high quality product.^1–3^ Anodes and cathodes are prepared by mixing and coating, and calendered to the required thickness and porosity. The electrodes are then combined with a liquid electrolyte and a porous, insulating separator, and sealed in the cell enclosure. Given the extended time required to make and test cells, it is important to characterise the intermediate components after each process step. For example, measurements of coating adhesion inform the manufacturability and long term stability of the coatings.^4^ Measurements of the electronic resistivity of the coatings are used to maximise the capacity at high rates of discharge. Typically, these use a four point probe to measure the in plane conductivity, in the *X* − *Y* direction. However, the actual direction of current flow during cell operation is in the through plane or *Z* direction. Thus, if the electronic conductivity is anisotropic, the wrong properties are being optimised.

**Fig. 1:**
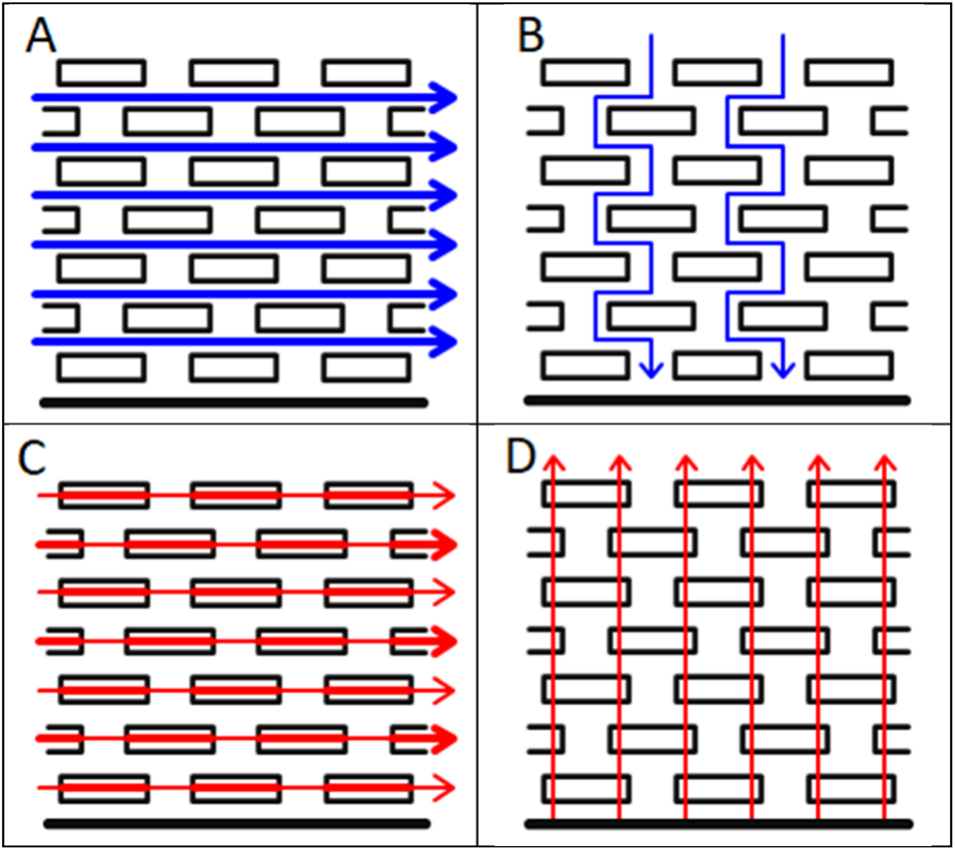
Anisotropy in tortuosity and electrical conductivity, (A) in plane tortuosity, (B) through plane tortuosity, (C) in plane conductivity and (D) through plane conductivity.

Standard lithium ion electrodes are composites of active materials, polymeric binders, and conductive carbon, coated onto thin metal foils. As such, heterogeneities are expected at the microscopic level. However, the morphology of the active material particles, and the electrode fabrication techniques, can lead to more systematic, larger scale differences in properties, which can cause anisotropy. A well known example is tortuosity in graphite platelet electrodes,^5^ as shown schematically in Fig. 1. During the coating and drying process, the platelets tend to orientate themselves parallel to the metal foil. In consequence, the tortuosity is much higher in the through plane direction, which is the more important direction during the operation of the electrode. Similar anisotropy might be expected in the electrical conductivity, given that the difference between the in plane and through plane electronic conductivity in graphite single crystals is a factor of around 10 000.^6^ The conductive path between the particles, in all directions, is provided by the conductive carbon additive.

As well as *XY vs. Z* anisotropy, there can also be *X vs. Y* anisotropy. This is most likely in coatings containing fibrous materials *e.g.* carbon nano-tubes. Anisotropic resistivity has been measured in many geological and biological systems, but the closest analogy to lithium ion electrodes is probably polymer composites with conductive additives.^7–10^ Some examples of resistivity measurements on this type of sample are collected in Table 1. The composites studied were nickel coated graphite (NCG) fibres in polypropylene (PP),^8^ multiwall carbon nano-tubes (MWCNT) in a polyethylene (PE)/polycarbonate (PC) blend,^9^ and carbon black (CB) in a PE/polyethylene terephthalate (PET) blend.^10^ For some samples, measurements were made in three directions, for others just parallel and perpendicular to the direction of extrusion. As might be expected, the fabrication method had a strong influence on the alignment of the fibres, and hence the resistivity values. However, even with spherical carbon black particles, there was a significant degree of anisotropy.

**Table 1:** Anisotropic resistivity measurements on polymer composites

Polymer	Additive	Fabrication method	Volume resistivity (Ω cm)		
			Longitudinal	Transverse	Normal
PP	4% NCG fibres	Compression	5.0	9.5	10.5
PP	4% NCG fibres	Extrusion	1.0	5.0	18.0
PP	4% NCG fibres	Injection moulded	1.0	5.0	15.0
PE/PC	4.5% MWCNT	Extrude + stretch	6 × 10^5^		5 × 10^6^
PE/PET	5% CB	Extrude + stretch	5 × 10^3^		2 × 10^5^

Electronic and ionic conductivity are both important parameters in achieving full utilisation of all the active material in the electrode. They are particularly important in electrodes intended for high power cells, and in the increasingly thick electrodes being used in high energy cells. A recent review has considered all aspects of electrical conductivity in lithium ion electrodes, including materials, mechanisms, experimental techniques, and simulation.^11^ Electronic conductivity also becomes more important as the proportion of active material in the coating increases. It is not a limiting factor in the ubiquitous academic active : binder : carbon formulation of 80 : 10 : 10 wt%.^12^ It is potentially more important in 96 : 2 : 2 electrodes, and certainly in 98.5 : 1 : 0.5. Therefore, it is important to have a quick, accurate and meaningful measurement of electronic resistivity, during any cell development programme. Historically, the standard approach has been to use a four point probe, with two current probes, and two voltage measurement probes, a known distance apart. One problem with this technique is that it requires a special coating on an insulating substrate, which may not coat, dry or calender the same as the regular coating on metal foil. In addition, the probe measures the in plane resistivity, which may not be the same as the more important through plane resistivity.

An alternative four line probe was developed, based on 10 μm wide strips of nickel on a silica substrate, electroplated with copper and then gold.^13,14^ The probe was operated in two modes, to enable in plane and through plane resistance measurements. This allowed samples coated on metal foils to be characterised. Analysis of the data required the use of a model, operated in either 2D or 3D mode. The volume conductivity was assumed to be isotropic, even though, “it is likely that typical battery films exhibit some anisotropy in conductivity, due to non-spherical particles and directional fabrication steps.^13^” This approach has also been extended to a ring electrode system.^15^

As an alternative to four point probes, a two point probe has been developed, to measure the total resistance through the coating structure.^16^ Tests on NMC-111 cathodes showed an increase in relative resistivity after light calendering, but a net decrease after heavy calendering. This was attributed to breaking conduction pathways initially, followed by re-establishing them with a shorter path length. For graphite anodes, calendering reduced the long range conductivity pathways, and increased the resistivity. The same approach has recently been used to compare single layer and double layer cathodes.^17^

**Fig. 2:**
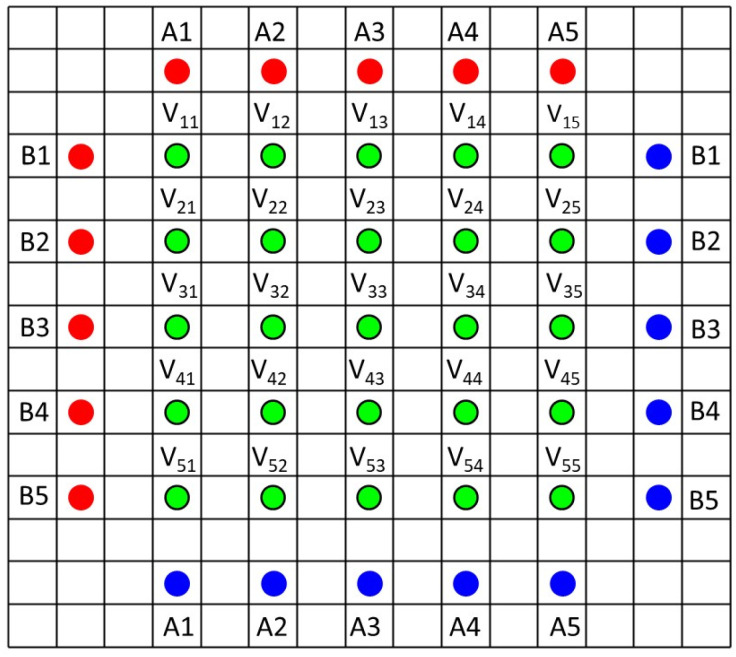
Pin arrangement of Hioki resistance measurement system.

Recently, a resistance measurement device has been introduced by Hioki, with a 46 point probe.^18,19^ It uses a 5 × 5 grid of voltage sense probes in the centre, and an array of current source and drain probes outside. The pin arrangement is illustrated in Fig. 2 (the 46th pin is used for probe orientation purposes). During operation, voltage measurements are made with ten different current configurations (A1–A5 and B1–B5). A finite volume model (FVM) is then used to calculate the volume resistivity of the coating, and the interface resistance between the coating and the foil. Hence, the system uses actual coatings, rather than special coatings on insulating substrates, and can also characterise each side of double sided coatings. The system is supplied with a finite volume model, to extract the resistance values. A minor issue is that the model takes several minutes to run, on its higher precision settings. More significantly, the model assumes that the volume resistivity of the coating is isotropic. The instrument has already been used in a number of papers,^20–23^ and is also used for quality control purposes during electrode manufacturing. Data from the 46 point probe was compared with the results from two point probe and impedance measurements, on various cathode coatings.^23^

For lithium ion battery electrodes, we expect anisotropy in thermal conductivity,^24,25^ mechanical properties like expansion and contraction, and the resultant stresses and strains,^26^ and as already noted, tortuosity.^5^ For electrical conductivity, there is an important difference between anodes and cathodes. In cathodes, the active materials have low or very low electronic conductivities, and conductivity is dominated by the conductive carbon additive.^27^ Therefore, the electronic conductivity depends on the distribution of the carbon binder domains. In anodes, graphite and (doped) silicon have much higher electronic conductivities than any of the standard cathode materials, and thus contribute to the electronic conduction pathways within the electrode structure.

The influence of formulation on various electrode properties including electronic conductivity was studied in NCA : PVDF : AB cathodes^28^ (NCA = LiNi_0.8_Co_0.15_Al_0.05_O_2_). The acetylene black : PVDF binder ratio needed to be at least 20%, to achieve decent conductivity. In further tests, a maximum conductivity was measured for an 80% ratio, but only in coatings with an impractically low active material content.^29^

The four line probe approach was used to measure the conductivity of commercial and laboratory made cathodes, with LCO (LiCoO_2_) as the active material.^30^ The conductivity increased as the porosity was reduced from 50% to 35%, and was then more or less constant down to 20%. The conductivity also increased as the acetylene black content was increased from 2 wt% to 4 wt% and then 6 wt%. In later studies, changes to electronic conductivity after cycling were measured for four types of electrode; LCO, LFP (LiFePO_4_) and NMC (LiNi_1−*x*−*y*_Mn_*x*_Co_*y*_O_2_) cathodes, and graphite anodes.^31^ Cycling caused a significant decrease in conductivity for the LCO and graphite electrodes, a smaller decrease for the NMC cathode, and a marginal increase for the LFP cathode. The data analysis also extracted values for the contact resistance between the coating and the current collector. These increased significantly with cycling for the LCO cathode and graphite anode, less for the NMC cathode, and only slightly (from a high initial value) for the LFP cathode. The electronic conductivity of an NMC-111 cathode increased as the porosity was reduced from 50% (uncalendered) to 40% and then 30%.^32^ The coating formulation was NMC-811 : PVDF : AB : SFG-6 = 81.6 : 8.0 : 6.4 : 4.0 wt%. When the conductivities were modelled using a dynamic collision model, it was necessary to use multiple layers with different porosities to match the experimental results.

A multi-layer model was also used to analyse conductivity data from LFP coatings with different carbon black (3, 6 or 10 wt%) and graphite (0 or 6 wt%) contents.^33^ Each layer was considered to be homogeneous and isotropic. Increasing the carbon black content reduced the volumetric resistivity, but increased the contact resistance between the coating and the carbon-coated aluminium foil.

The electronic conductivity of NMC-811 cathodes containing various amounts of different conductive carbons was measured in both the in plane and through plane directions.^34^ The electrodes contained 10 wt% PVDF, and either carbon black (0.1–20 wt%), graphene (0.1–30 wt%) or carbon nano-tubes (0.01–4 wt%). The mixes were coated on aluminium or glass slides, for the through plane and in plane measurements respectively. The glass slide implies that the coatings were not calendered, and also that the two coatings may have different morphologies. For each of the three carbon additives, there was a threshold mass or volume fraction, above which the conductivity increased with added carbon. At this threshold point, the in plane conductivity was 100 times higher than the through plane conductivity for carbon black, and 1000 times higher for the carbon nano-tubes. Rate tests suggested that the performance was limited by the electronic conductivity in the through plane direction.

The distribution of the carbon binder domain in cathodes can be measured experimentally using techniques like X-ray tomography. For a NMC-333 : PVDF : C65 = 92 : 4 : 4 wt% coating, the through plane conductivity was calculated to be slightly lower than the in plane conductivity.^27^ Interestingly, a finite element model (FEM) and a finite volume model (FVM) gave different results when applied to the same experimental data. Tomography data has also been used as the basis for modelling the distribution of the carbon binder domain (CBD) amidst the active material particles, and calculating the resultant electronic conductivity, tortuosity, and area available for electrochemical reactions.^35^ A morphology parameter was introduced, to quantify the tendency of the CBD to form layers or clumps. CBD layers reduced the electrochemical reaction area, whereas the greatest influence on tortuosity was the overall porosity. Electronic conductivity was only an issue at very high active material loadings.

There are far fewer studies on the electronic conductivity of lithium ion anodes. Open source X-ray tomography data on four graphite anodes was reanalysed, to look at the heterogeneity and anisotropy of porosity, tortuosity and conductivity.^36^ For each parameter, the anisotropy was calculated from the tomography data, and predicted from the structural anisotropy of the same volume. For conductivity, there was good agreement between the calculated and predicted anisotropies, in all three directions. The same tomography data was also analysed to look for inhomogeneities in the anode coatings, over multiple length scales.^37^ Graphite : CMC : SBR : CB = 96 : 1.5 : 1.5 : 1 wt% slurries were mixed with different solids contents during the kneading step, and with or without a pre-mix step.^22^ This caused differences in coating morphology, and the resistance values measured using a Hioki RM2610. The volumetric resistivities increased and then decreased, as the coating porosity was reduced. The interface resistivities decreased as the porosity was reduced.

The Nextrode project, funded by the Faraday Institution, is exploring various aspects of lithium ion cell manufacture. It is intended to be complementary to the DaLion projects at Braunschweig University,^38^ the Artistic project at the Université de Picardie Jules Verne,^39^ and the European Defacto project.^40^ One part of the Nextrode project is using a Design of Experiments (DoE) approach to investigate electrode manufacturing.^41,42^ The most recent DoE has looked at the formulation of cathode mixes, with LFP as the active material.^43,44^ The previous DoE investigated different calendering pressures and temperatures on graphite anodes and NMC-622 cathodes, prepared at different coat weights. Coatings from both these experiments have now been tested using the Hioki RM2610, to provide the experimental results for this report. Simultaneously, a Comsol Multiphysics® model has been developed for the test arrangement, to help interpret the data.

## Results and discussion

### Experimental measurements

**Fig. 3:**
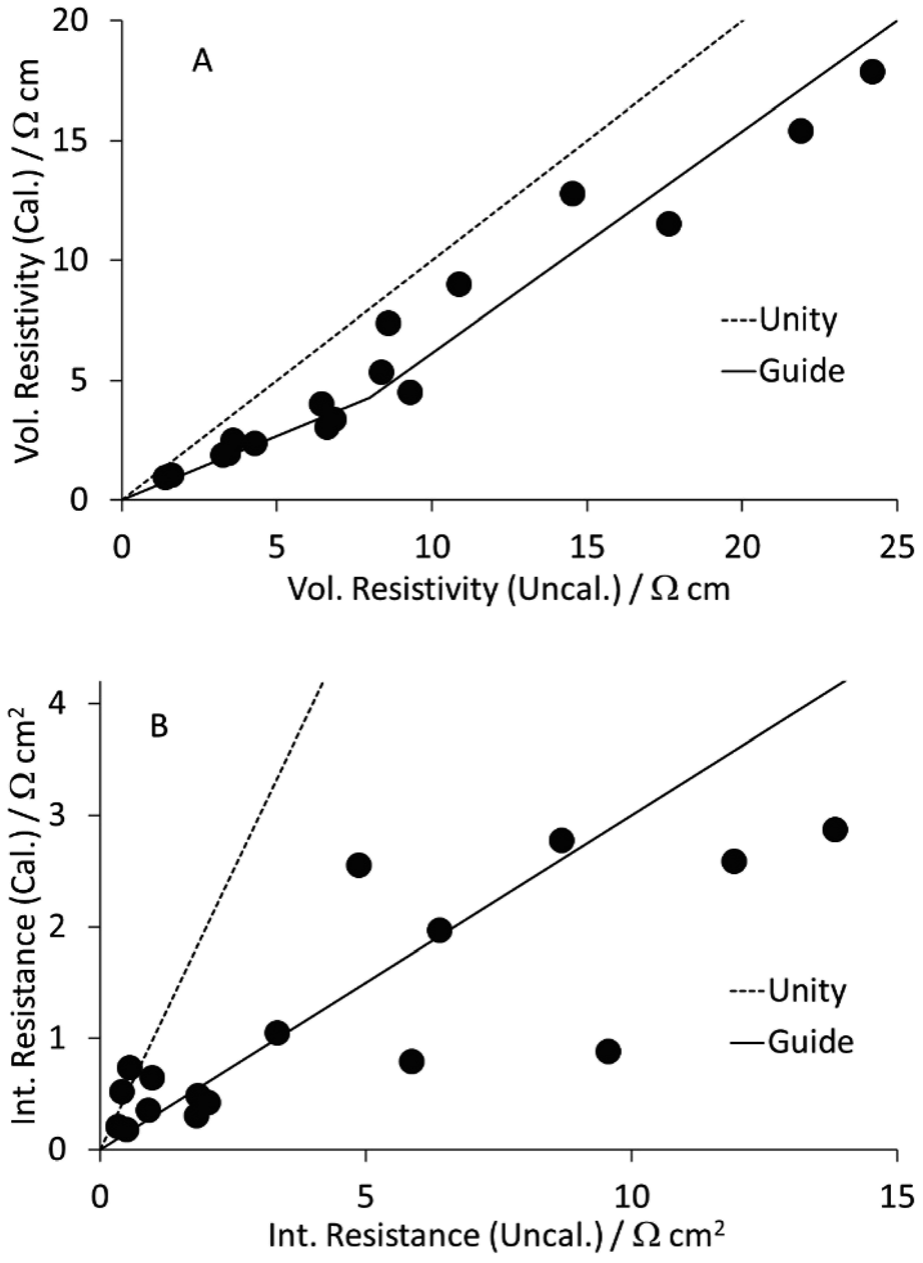
Effect of calendering LFP cathodes on (A) volumetric resistivity and (B) interface resistance.

**Fig. 4:**
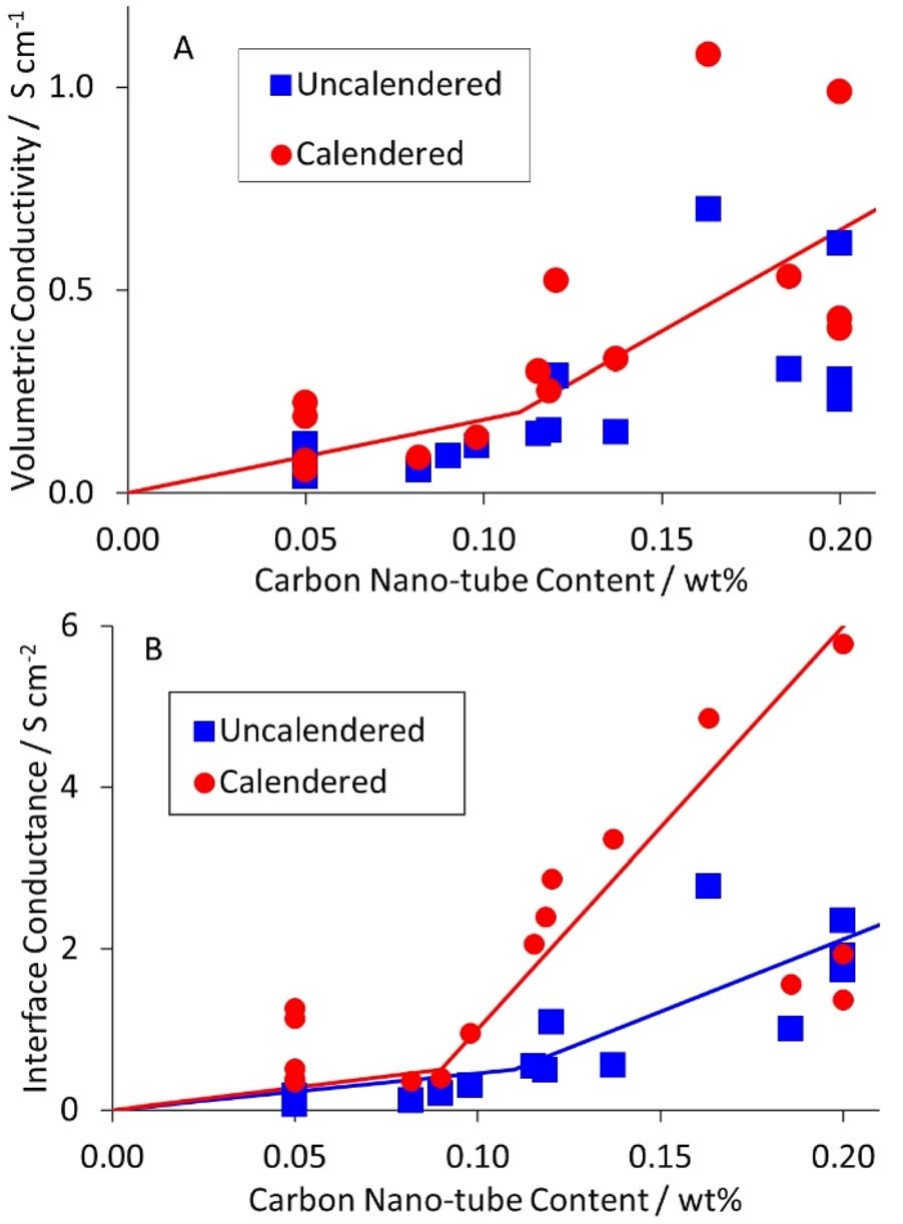
Influence of carbon nano-tube content in LFP cathode on (A) volumetric conductivity, and (B) interface conductance.

As part of a DoE on LFP formulations,^43^ a set of seventeen mixes were prepared and coated. Subsequently, a further mix was prepared using the same approach, as part of a scale up activity.^44^ The formulations are listed in Table S1 in the ESI.† The KS6L graphite component has a D_50_ of 3.4 μm. The SWCNT have diameters of 1.6 nm and lengths of 5+ μm, giving a length: diameter ratio of >3000. The coatings were used for electrochemical tests in coin cell half cells, for adhesion tests, and for the measurement of electronic resistivity, using the Hioki RM2610. The results from the latter test are tabulated in Table S6,† and illustrated in Fig. 3. The dotted lines are for unity. The solid lines in this (and subsequent) figures are guides to the general trend. Each value is an average of five measurements, made in different areas of a piece of coating. After calendering, the volumetric resistivities were slightly lower, and the interface resistances often significantly lower. Full consideration of the DoE involves multiple input and output parameters, in a multi-variate analysis. However, even a simple one to one analysis of the resistivity values *versus* the carbon nano-tube content in the mix showed some obvious trends. These are illustrated in Fig. 4. Both the volumetric conductivities and interface conductances increased with a higher carbon nano-tube content, as might be expected.

All the data plotted in Fig. 3 and 4 used the Hioki model with area = medium and element size = super fine. Values for one data set using all nine Hioki models are collected in Table S3.† The values obtained depended on the model, particularly the element size. For the super fine element size, the difference between area = medium and area = wide did not justify the extra calculation time. Even so, the total calculation time for all 170 samples was 55 hours. The distribution of calculation times using both the normal/normal and super fine/medium models is plotted in Fig. S1.† For this sample set, the modelling time was shorter for the calendered than the uncalendered coatings, for both models.

A previous DoE had investigated different calendering conditions on a graphite anode and an NMC-622 cathode.^45^ The formulations for these two coatings are summarised in Table S2,† as anode #1 and cathode #1. The graphite particles had a D_50_ = 15.6 μm and were irregularly shaped *i.e.* neither spherical nor platelet. The carbon black in the cathode had a particle size of ∼50 nm, and clumped in irregular “lacy” networks. Each coating was prepared at two different coat weights; light (2.0–2.2 mA h cm^−2^) and heavy (3.0–3.3 mA h cm^−2^). Each was calendered at three different temperatures, and to three target porosities. Some of these samples were still available for resistivity measurements.

**Fig. 5:**
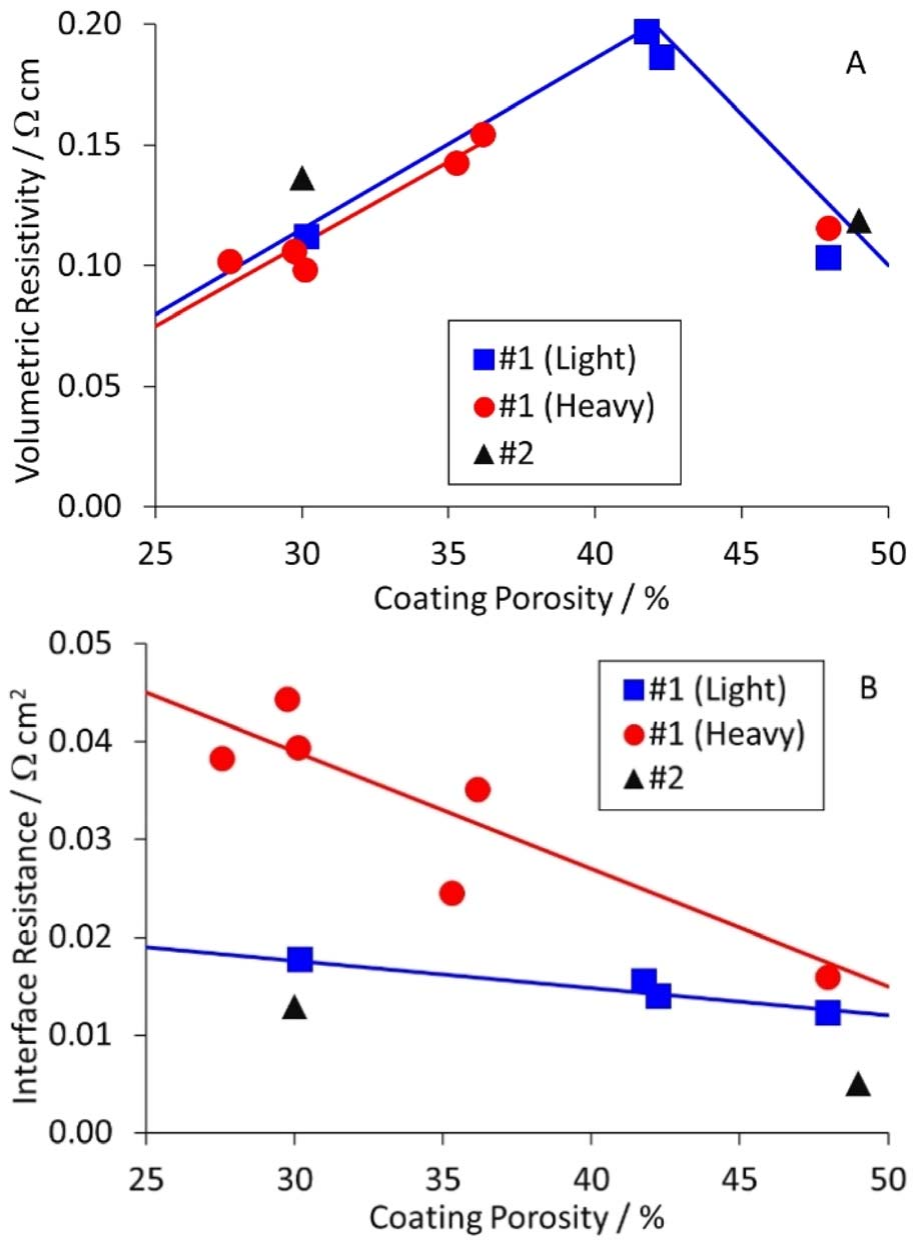
Effect of anode coating porosity on (A) volumetric resistivity and (B) interface resistance.

The results for the anode are collected in Table S7,† and plotted in Fig. 5. The table and figure also include results for anode #2, which was prepared as part of a DoE on coating parameters. The results were interesting, and perhaps unexpected. The volumetric resistivities increased for light calendering, and then decreased to the original values for heavier calendering. The interface resistances increased as the porosity decreased, particularly for the heavier coat weight materials. The actual and expected changes to resistivity with calendering are discussed later in this document.

**Fig. 6:**
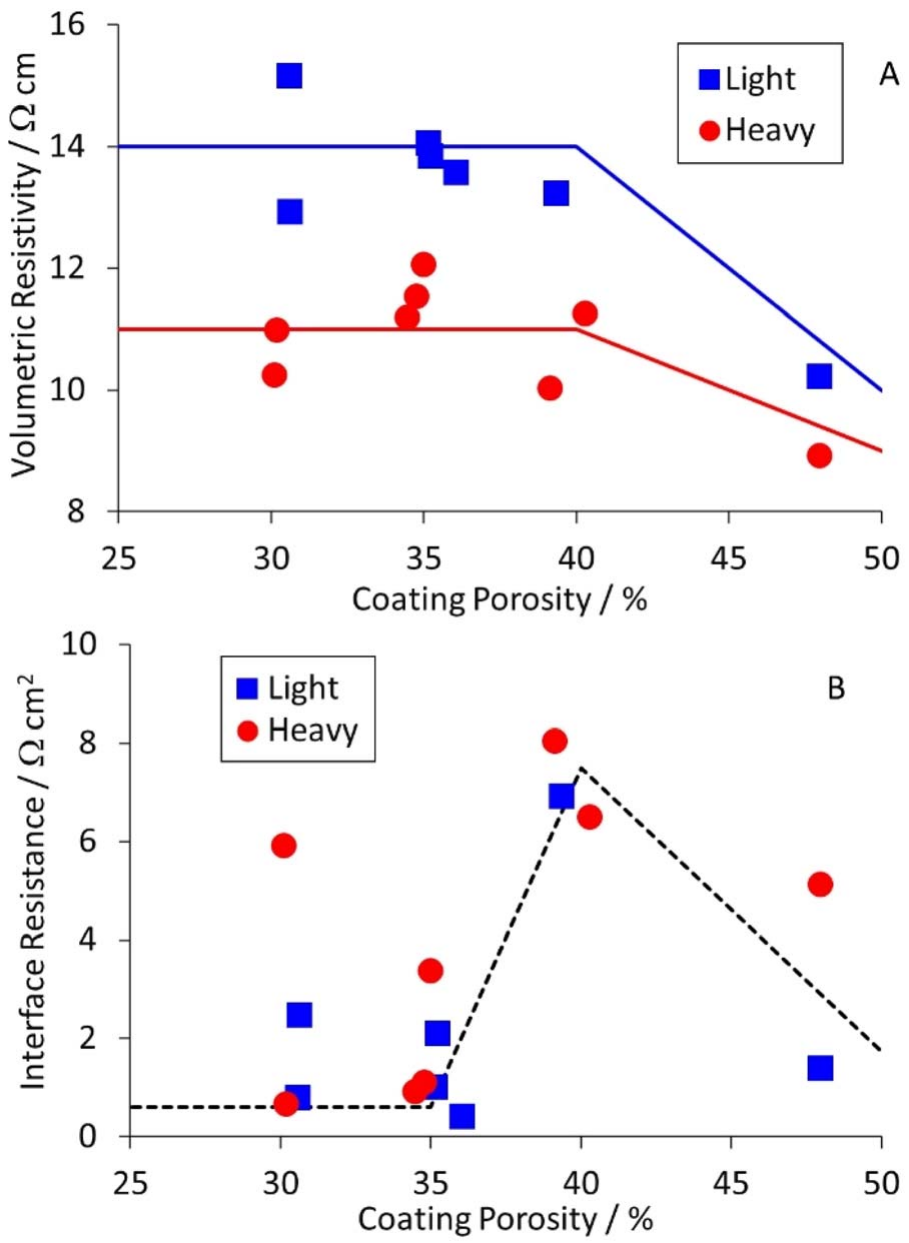
Effect of cathode coating porosity on (A) volumetric resistivity and (B) interface resistance.

Equivalent results for the NMC-622 cathodes are collected in Table S8,† and plotted in Fig. 6. The volumetric resistivities for both coat weights increased after light calendering, but then showed little further change. The interface resistances increased for light calendering, and then dropped again as the porosity was reduced further. The implication is that heavier calendering forces the active material particles through the native alumina layer on the aluminium foil, and significantly reduces the interface resistance.

### Data analysis and anisotropy

As with the LFP cathode coatings, results from all nine Hioki models on one data sample are collected in Tables S4 and S5.† All the data in Fig. 5 and 6 was for the area = medium, element size = super fine model. The total times to model 29 anode measurements and 69 cathode measurements were three and twenty hours, respectively. One feature that is often requested in finite element and finite volume models is “mesh independence” *i.e.* the results do not depend on the size of the mesh volume. Clearly, the nine Hioki models do not achieve mesh independence.

All the data was analysed with two models; normal/normal and medium/super fine. The calculated values from the two models for the volumetric resistivities and interface resistances are plotted in Fig. S2 and S3.† The volumetric resistivity plots were generally linear, with a slope of just less than unity. However, for the interface resistances, the graphs usually had two regions, with different slopes. In most cases, the slope at high values was more gentle, but it was steeper for the uncalendered LFP coatings. It is unclear if this is a genuine effect, or a modelling artefact.

Calendering creates a uniform surface on the coating, and reduces the coating thickness and the overall coating porosity. However, should we expect the volumetric resistivity to increase, decrease or stay the same after calendering? It is possible to envisage three scenarios:-

• Calendering creates extra conduction pathways in the coating.

• Calendering does not change the conduction pathways in the coating.

• Calendering breaks conduction pathways in the coating.

#1 would be expected to reduce the coating resistance, #2 to keep it the same, and #3 to increase it. By definition, the resistivity (Ω cm) is equal to the resistance (Ω) × area (cm^2^)/length (cm). If the resistance remains the same after calendering (#2), and the area decreases, then the resistivity should decrease. If the resistivity increases after calendering, the implication is that more conduction pathways have been broken than created. For practical electrodes, the decrease in the interface resistance after calendering may be more important than any increase in volumetric resistivity. The reduction in thickness after calendering will usually decrease the total through plane resistance, even if the volumetric resistivity has increased slightly. For a 100 μm thick coating with a volumetric resistivity of 10 Ω cm and an interface resistance of 1 Ω cm^2^, the total through plane resistance = 1.0 + 10 × 0.01 = 1.1 Ω cm^2^.

Previous measurements using a two pin probe to measure the total through coating resistance, showed an increase and then decrease with reducing porosity for cathodes, and a steady increase for anodes.^16^ This matches the interface resistance plots shown in Fig. 5 and 6. Since the interface resistance contributes around 90% of the total through coating resistance for these samples, the trends are consistent. Measurements of volumetric resistivity *vs.* porosity on a similar anode mix showed a similar maximum to that in Fig. 5A.^22^ However, the interface resistances decreased with reduced porosity, unlike Fig. 5B. There are a number of possible explanations for this difference; the morphology of the graphite particles, the surface finish of the copper foil, or the “power” of the calendering equipment.

**Fig. 7:**
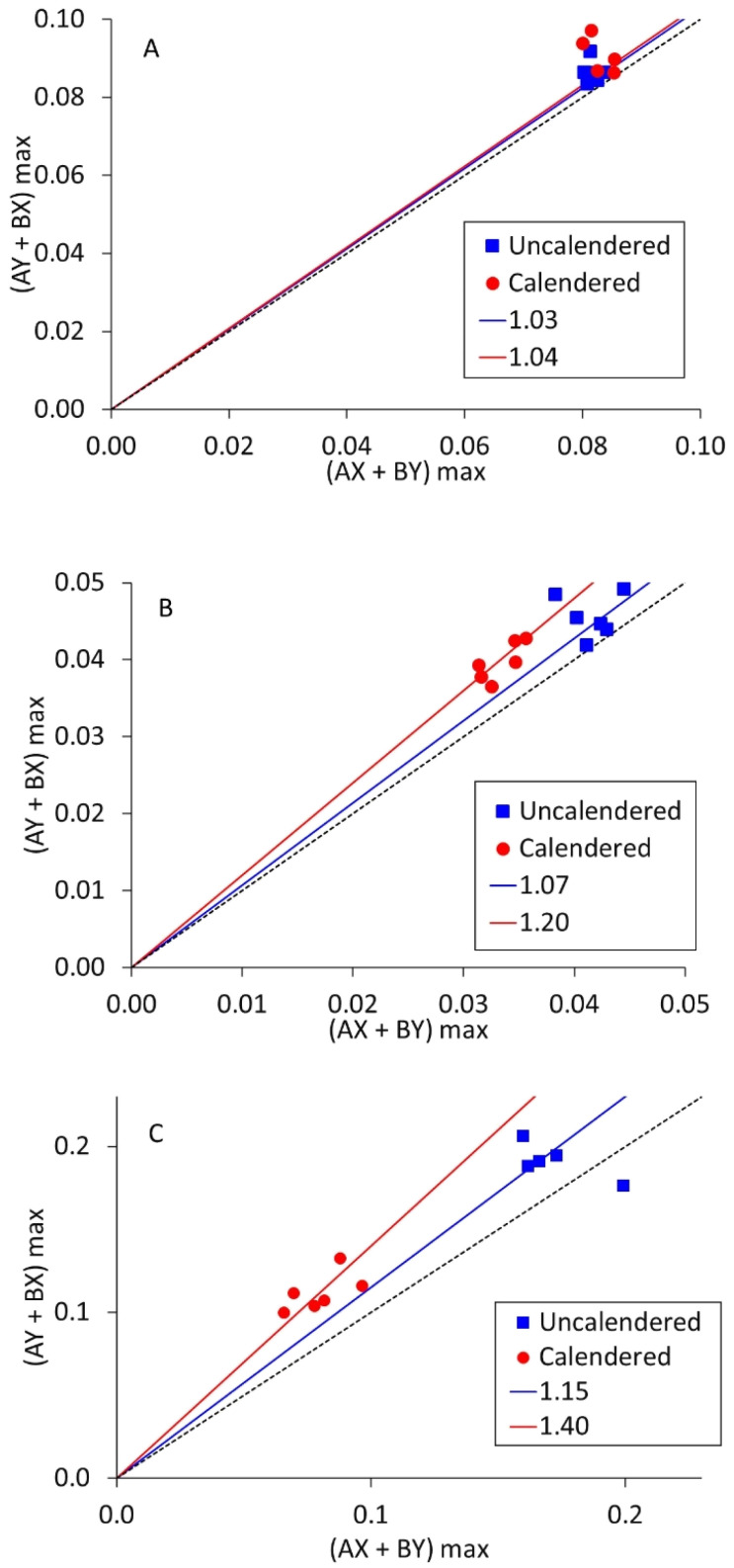
*X*–*Y* anisotropy factor measurements on LFP cathodes, for (A) mix 14, 0.05 wt% SWCNT, (B) mix 11, 0.16 wt% SWCNT, and (C) mix 18, 0.16 wt% SWCNT.

As mentioned in the introduction, each measurement collects data from ten different combinations of current source and drain. Five are in the ‘*A*’ direction, and five in the perpendicular ‘*B*’ direction. Resistivity measurements on polymer composites containing carbon fibres have shown anisotropy, if the fibres become aligned during the fabrication process. To investigate this, measurements were deliberately made with LFP coatings in two different orientations, designated ‘*X*’ and ‘*Y*’. If there is *X*–*Y* anisotropy, then the maximum voltages in the *AX*/*BY* directions will be different from the *AY*/*BX* directions. These two voltages are identified as an *X*–*Y* anisotropy factor in Fig. 7, for three LFP coatings. The gradients for the black dotted lines are 1.0; the gradients of the other lines are included in the figures.

It is evident that most of the points in Fig. 7 are above the line of unity. However, Fig. S4† shows equivalent plots for the other three coatings tested here. In each case, the data was effectively on the line of unity. Therefore, *X* – *Y* anisotropy in volumetric resistivity can occur, but not in every coating. Comparing the degree of anisotropy (slope gradient) with the SWCNT content, mixes 11 and 18 contained more SWCNT and produced steeper gradients than mix 14. Mix 11 contained more PVDF binder than mix 18, leading to a higher mix viscosity. Thus, as a first order effect, more SWCNT leads to more anisotropy, but the other components in the mix also have an influence. Calendering occurs in the same direction as coating, and in each case there was more anisotropy after calendering.

### Comsol modelling

The original aspiration of the Comsol^46^ modelling activity was to develop a “script” model, which could be used to process data from the Hioki instrument. It soon became evident that this would be extremely difficult. Experimentally, it was clear that the applied current scaled by a factor of ten, to match the resistivity of the sample. Measurements were made on a sample containing a 108 μm thick aluminium foil on a 123 μm copper foil. Since the resistivity of these metals is known, the applied current could be adjusted to replicate the measured voltage values. The experimental and model voltages are collected in Table S11.†

**Fig. 8:**
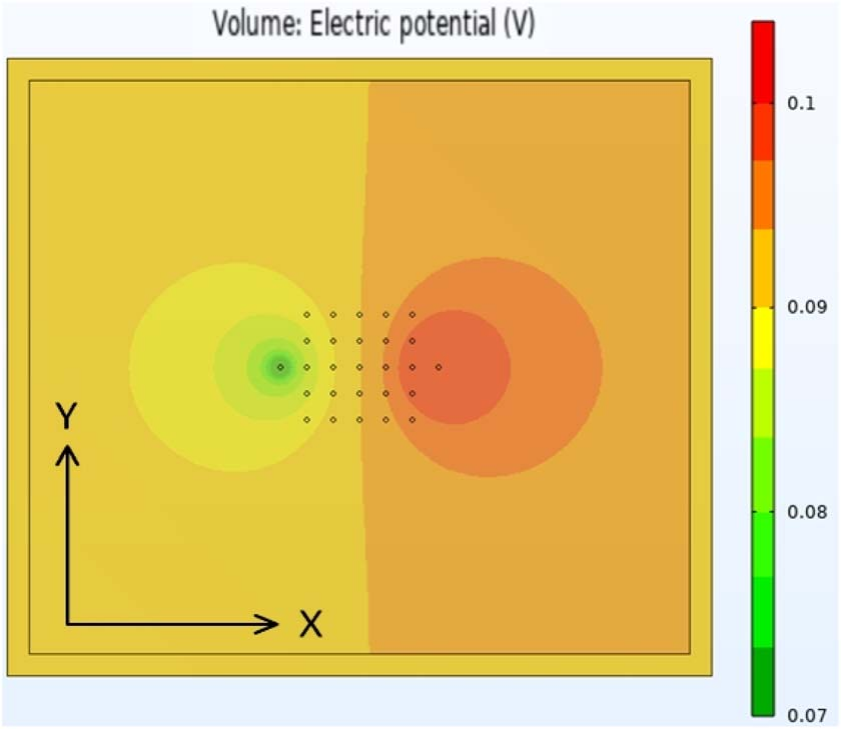
Voltage distribution map for coating model during Hioki RM2610 test.

After replicating the physical geometry of a sample being tested by the Hioki RM2610, the model was run using parameters for a generic anode coating on copper foil. Most of the input parameters were fixed, but some were varied systematically. The default values for these parameters are listed in Table S12.†Fig. 8 shows the calculated voltage distribution across the sample, for the default parameters. The voltages at the 25 voltage pins were extracted from the complete data set, and used to calculate the maximum voltage drop across the pins. Fig. S5† shows the influence of the different input parameters on this maximum voltage. Qualitatively, the results were in line with expectations. The maximum voltage drop increased with increasing coating resistivity, interface resistance, and applied current, and increased with reduced coating thickness and modelled area. The model was also run with four meshing combinations, defined by the parameters in Table S13.† The results were effectively mesh independent.

The model was then converted to a generic cathode coating with anisotropic conductivity, on aluminium foil with different thicknesses. A diagonal conductivity matrix was used, using values for {*σ*_*X*_, *σ*_*Y*_, *σ*_*Z*_}. Maximum voltage values were calculated for different foil thicknesses and conductivity values, and are collected in Table S14.†

It was evident that the voltages changed significantly with conductivity, but minimally with foil thickness. Fig. S6† plots calculated current densities in the *X* and *Z* directions, for different conductivity matrices and foil thickness values. Reducing the *Z* direction conductivity reduced the current flow down into the foil, but the interface resistance between the coating and the foil had a much greater impact than the total foil resistance.

Initially, it was hoped that measurements with different foil thicknesses could be used to investigate any *XY vs. Z* anisotropy. The modelling results showed that any changes due to foil thickness would be much smaller than the observed variations in experimental measurements. Typically, the spread in volumetric resistivity values was around ±10%; outlying results of ±20% were excluded from the averaging process. The raw voltage data files showed variations between the ten measurements, and occasional stray values. This is to be expected in samples with microscopic heterogeneity, and a rough surface. Clearly the Hioki model has to cope with variability and spread in the raw data. However, this could be reduced if it used *X* and *Y* conductivity values, rather than assuming isotropic conductivity. On the higher resolution settings, the model often failed to find a suitably accurate solution, within the designated 30 iterations.

With regards to measurements of *XY vs. Z* anisotropy on thin films, the best technique seems to be using four point probes with significantly different spacings.^47^ There is a transition between 3D current flow (spacing ≪ film thickness) to 2D current flow (spacing ≫ film thickness). The transition point changes between isotropic and anisotropic coatings, and the data can be fitted to obtain *ρ*_*X*_ and *ρ*_*Z*_.

## Methodology

The LFP cathodes were prepared using a Thinky® planetary mixer, and then coated onto aluminium foil using an Erichsen draw down coater. The coating solvent was NMP (*n*-methyl pyrrolidinone). The graphite anode and NMC-622 cathode mixes were prepared using a one litre Eirich mixer, and coated using a Megtec reel to reel coater. Where required, coatings were calendered using an Innovative Machines Corp. sheet calender.

The electronic resistivity measurements were made using a Hioki RM2610 instrument. The instrument has four measurement speeds; all the tests reported here used the “medium” speed setting. The quickest analysis model was run after each test, to check the validity of the data. Subsequently, one or more higher precision models were run on the data. All the tests were performed in an air conditioned laboratory. The temperature measurements on the Hioki sensor were typically 20 ± 1 °C. To change the current distribution in some of the test samples, extra tests were performed with a thin metal sheet under the coating. This was 123 μm copper for the anodes, and 108 μm aluminium for the cathodes. Although thin, these sheets were significantly thicker than the 10–15 μm foils used to coat the electrodes.

**Fig. 9:**
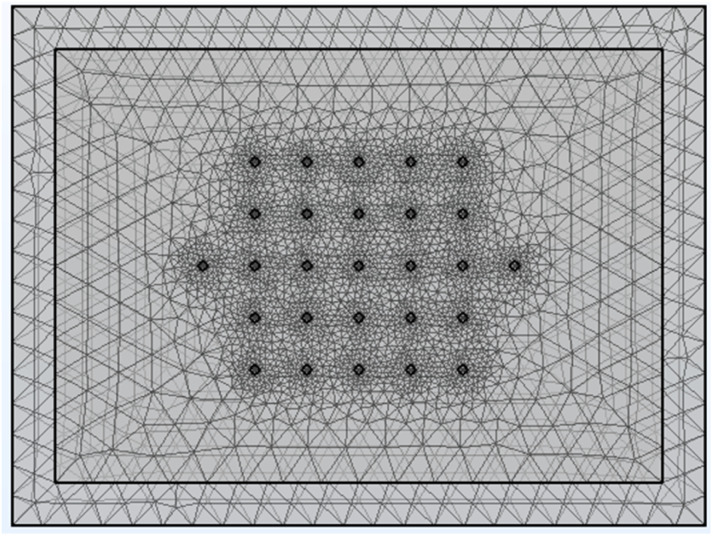
Typical model meshing arrangement for thin foil, coating, and 27 measurement pins.

The Comsol Multiphysics® model contained a block of coating material, a thin sheet of metal foil (aluminium or copper), and 27 small cylinders. These represent the 25 voltage sense pins, and two of the current pins (A3 and B3 test configurations). One current pin was designated as a current source, and the other as a ground. All 27 cylinders had a diameter of 20 μm, a height of 2 μm, and a spacing of 120 μm. Details of the model are set out in Tables S9 and S10.†Fig. 9 shows an example of the sample meshing produced by the model. The coating resistivity, the interface resistance between the coating and the foil, and the applied current were treated as input variables. The main output values were the voltages at the surface on the coating, adjacent to the 25 voltage sense pins.

## Conclusions

The results described in this paper lead to two important conclusions:

• The volumetric conductivity of lithium ion electrodes can be anisotropic in the *X vs. Y* directions, and may well be anisotropic in the *XY vs. Z* directions as well. Thus any electrode optimisation work based on in plane conductivity measurements is potentially wrong. Four point probe measurements on circular discs cut from electrodes should use a consistent probe alignment with the coating direction. Any model that uses measured values for *σ*_*xy*_ as the value for parameter *σ*_*z*_ may need improved methods to measure the through plane conductivity of the coating.

• The main benefits of calendering have been assumed to be lower porosity (higher energy density), and a uniform surface finish. However, for cathodes, there is also a significant reduction in the interface resistance after calendering, which will increase the capacity and improve rate performance.

Anisotropy is most likely to occur when the mix contains a component like single wall or multi wall carbon nano-tubes. The fibres tend to align during coating, and this can be reinforced during the subsequent calendering step. It was not possible to measure any *XY vs. Z* anisotropy using the Hioki instrument; this requires multiple probes with a wide range of spacings.^47^

An important advantage of using the Hioki RM2610 device is that it operates on regular coatings, on thin metal foils, rather than requiring special coatings on insulating substrates. Some of the other observations drawn from the measurements:-

• The total through plane resistance is usually dominated by the interface resistance between the coating and the foil, rather than the volumetric resistivity of the coating.

• The interface resistance for cathodes decreases significantly after calendering, as the active material particles are forced through the native oxide layer on the aluminium.

• For NMC-622 cathodes calendered to different porosities, the interface resistance increased and then decreased, as the porosity is decreased.

• For graphite anodes, the interface resistance increased slightly as the porosity is decreased, but the volumetric resistance increased and then decreased again.

• For LFP cathodes, the volumetric resistivity and interface resistance both decreased with increased carbon nano-tube content in the formulation.

The instrument uses a finite volume model to calculate volumetric resistivity and interface resistance values. A similar model was created in Comsol Multiphysics®, to help interpret the data. The Comsol model proved that it was not possible to measure *XY vs. Z* anisotropy with the Hioki instrument. Any model that processes the Hioki data must cope with the inevitable variability when measuring samples with microscopic heterogeneities in their structure. The model would benefit from being able to use different values for the conductivities in the *X* and *Y* directions.

## Author contributions

Conceptualisation (MJL), investigation (GA, DED, MJL), formal analysis (MJL), software (DW, MJL), writing – original draft (MJL), writing – review & editing (all), funding acquisition (JM, MC).

## Conflicts of interest

There are no conflicts to declare.

## Supplementary Material

RA-013-D3RA06412C-s001
