# Supplementary material for: Measurement of anisotropic volumetric resistivity in lithium ion electrodes
Source: RSC Adv. 2023 Nov 14;13(47):33437–45. doi: 10.1039/d3ra06412c (PMC10644285; doi:10.1039/d3ra06412c)
Supplement: RA-013-D3RA06412C-s001 [file RA-013-D3RA06412C-s001.pdf]

Electronic Supplementary Material (ESI) for RSC Advances.
This journal is © The Royal Society of Chemistry 2023

Measurements Of Anisotropic Volumetric Resistivity In Lithium Ion Electrodes

Supplementary Information

Table S.1. LFP Electrode Compositions

Name	LFP / wt%	PVDF / wt%	KS6L / wt%	SWCNT / wt%	Thickness / um	
					Uncal.	Calendered
MIX01	96.98	0.93	1.91	0.19	143	83
MIX02	96.40	0.75	2.80	0.05	141	88
MIX03	95.32	1.76	2.80	0.12	150	85
MIX04	94.67	2.33	2.80	0.20	155	82
MIX05	96.49	1.97	1.49	0.05	147	86
MIX06	97.12	1.98	0.70	0.20	158	79
MIX07	96.20	1.85	1.83	0.12	166	77
MIX08	94.17	3.00	2.78	0.05	136	78
MIX09	98.47	0.75	0.70	0.08	150	83
MIX10	96.00	1.00	2.80	0.20	179	83
MIX11	94.91	3.00	1.92	0.16	159	84
MIX12	96.20	3.00	0.70	0.10	138	81
MIX13	96.84	1.30	1.75	0.12	164	92
MIX14	97.00	2.25	0.70	0.05	154	76
MIX15	96.27	1.84	1.75	0.14	160	76
MIX16	97.10	0.75	2.10	0.05	149	91
MIX17	95.86	1.25	2.80	0.09	149	88
MIX18	97.03	1.94	0.87	0.16	142	80
MIX21	97.03	1.94	0.87	0.16	148	80

Table S.2. Other Anode And Cathode Compositions

I.D.	Active	Binder	Carbon	Content / wt%		
				Active	Binder	Carbon
Anode #1	Graphite	CMC + SBR	C45	95.25	1.5+2.25	1.00
Anode #2	Graphite	CMC + SBR	C45	95.25	1.5+2.25	1.00
Cathode #1	NMC-622	PVDF	C65	96.00	2.00	2.00

Table S.3. Hioki Model Results For LFP Cathode Coating (Mix11C)

Element size	Model area	Calculation / h:m:s	Iterations	Coincidence	Resistivity / Ω cm	Interface / Ω cm²	Surface / mΩ cm²
Normal	Normal	00:00:08	11	0.9964	0.857	0.205	7.20
Normal	Medium	00:01:00	21	0.9957	0.830	0.274	6.97
Normal	Wide	00:01:36	16	0.9956	0.828	0.284	6.95
Fine	Normal	00:02:13	30	0.9976	0.898	0.175	7.54
Fine	Medium	00:06:17	19	0.9967	0.874	0.217	7.34
Fine	Wide	00:15:06	22	0.9966	0.872	0.222	7.33
Super Fine	Normal	00:02:41	9	0.9966	0.930	0.162	7.82
Super Fine	Medium	00:10:21	8	0.9956	0.907	0.194	7.62
Super Fine	Wide	00:58:30	30	0.9955	0.905	0.197	7.61

Table S.4. Hioki Model Results For Anode Coating #1

Element size	Model area	Calculation / h:m:s	Iterations	Coincidence	Resistivity / Ω cm	Interface / Ω cm²	Surface / mΩ cm²
Normal	Normal	00:00:04	8	0.9934	0.097	0.0132	0.602
Normal	Medium	00:00:14	7	0.9936	0.095	0.0143	0.589
Normal	Wide	00:00:25	6	0.9936	0.095	0.0143	0.589
Fine	Normal	00:00:30	7	0.9971	0.103	0.0117	0.636
Fine	Medium	00:01:48	7	0.9971	0.101	0.0123	0.626
Fine	Wide	00:03:31	7	0.9971	0.101	0.0124	0.626
Super Fine	Normal	00:01:39	7	0.9974	0.107	0.0111	0.662
Super Fine	Medium	00:05:51	7	0.9973	0.105	0.0116	0.653
Super Fine	Wide	00:12:01	7	0.9973	0.105	0.0116	0.653

Table S.5. Hioki Model Results For Cathode Coating #1

Element size	Model area	Calculation / h:m:s	Iterations	Coincidence	Resistivity / Ω cm	Interface / Ω cm²	Surface / mΩ cm²
Normal	Normal	00:00:10	11	0.9936	9.65	1.55	46.3
Normal	Medium	00:00:40	13	0.9938	9.46	1.65	45.4
Normal	Wide	00:01:16	12	0.9938	9.46	1.65	45.4
Fine	Normal	00:01:00	8	0.9988	10.2	1.37	49.1
Fine	Medium	00:03:38	7	0.9989	10.1	1.43	48.4
Fine	Wide	00:06:52	7	0.9989	10.1	1.44	48.4
Super Fine	Normal	00:03:10	8	0.9995	10.7	1.31	51.1
Super Fine	Medium	00:11:49	9	0.9995	10.5	1.36	50.5
Super Fine	Wide	00:20:45	9	0.9995	10.5	1.36	50.4

Table S.6. Calculated LFP Cathode Average Resistivities

Name	Volumetric Resistivity / Ω cm				Interface Resistance / Ω cm^2			
	Uncalendered		Calendered		Uncalendered		Calendered	
	Mean	S.D.	Mean	S.D.	Mean	S.D.	Mean	S.D.
MIX01	3.28	0.36	1.87	0.30	1.00	0.36	0.64	0.36
MIX02	9.32	0.66	4.49	0.34	9.59	0.62	0.88	0.11
MIX03	3.47	0.07	1.91	0.06	0.92	0.16	0.35	0.08
MIX04	1.63	0.17	1.01	0.08	0.52	0.28	0.17	0.03
MIX05	14.56	0.59	12.76	0.42	6.40	1.98	1.97	0.23
MIX06	3.60	0.47	2.47	0.30	0.58	0.18	0.73	0.55
MIX07	6.87	0.77	3.34	0.49	1.86	1.23	0.48	0.13
MIX08	8.39	0.65	5.31	0.62	5.88	1.91	0.79	0.12
MIX09	17.66	1.22	11.50	1.93	8.70	2.99	2.77	1.42
MIX10	4.32	0.32	2.33	0.29	0.43	0.16	0.52	0.25
MIX11	1.43	0.07	0.93	0.09	0.36	0.11	0.21	0.03
MIX12	8.62	0.23	7.35	0.65	3.35	0.49	1.05	0.25
MIX13	6.48	0.56	3.99	0.30	2.05	1.40	0.42	0.11
MIX14	24.22	0.98	17.83	0.98	13.85	3.01	2.87	0.52
MIX15	6.64	0.72	3.04	0.52	1.83	1.06	0.30	0.07
MIX16	21.93	0.64	15.39	0.88	11.95	1.74	2.58	0.78
MIX17	10.91	1.33	8.99	1.03	4.89	0.98	2.55	1.06
MIX18	5.36	0.20	3.90	0.34	1.22	0.11	0.10	0.01
MIX21	4.04	0.07	2.35	0.16	0.65	0.13	0.30	0.04

Table S.7. Calculated Anode Average Resistivities

Designation	Temp. / °C	Porosity / %	Volumetric Resistivity / Ω cm		Interface Resistance / Ω cm^2	
			Mean	S.D.	Mean	S.D.
L_Uncal	N / A	48.0	0.103	0.002	0.012	0.002
L_45_P	45	42.3	0.186	0.000	0.014	0.000
L_60_P	60	41.8	0.196	0.009	0.015	0.000
L_60_D	60	30.2	0.112	0.002	0.018	0.000
H_Uncal	N / A	48.0	0.115	0.005	0.016	0.001
H_RT_D	25	29.8	0.106	0.003	0.044	0.005
H_45_M	45	35.3	0.142	0.004	0.024	0.002
H_45_D	45	30.1	0.098	0.003	0.039	0.006
H_60_M	60	36.2	0.154	0.002	0.035	0.001
H_60_D	60	27.6	0.101	0.000	0.038	0.001
#2_U	N / A	~ 50	0.119	0.003	0.005	0.001
#2_C	R. T.	~ 30	0.136	0.004	0.013	0.001

Table S.8. Calculated Cathode #1 Average Resistivities

Designation	Temp. / °C	Porosity / %	Volumetric Resistivity / Ω cm		Interface Resistance / Ω cm^2	
			Mean	S.D.	Mean	S.D.
L_Uncal	N / A	**48.0**	10.21	0.24	1.369	0.146
L_85_M	85	36.1	13.57	1.40	0.404	0.109
L_120_M	120	35.2	13.84	1.13	2.079	1.783
L_120_D	120	30.6	12.91	0.20	2.456	1.406
L_145_P	145	39.4	13.22	0.47	6.919	1.077
L_145_M	145	35.2	14.05	0.94	0.998	0.052
L_145_D	145	30.6	15.15	0.11	0.781	0.105
H_Uncal	N / A	**48.0**	8.91	0.12	5.110	0.502
H_85_P	85	40.3	11.24	1.93	6.485	4.905
H_85_M	85	34.5	11.18	0.38	0.907	0.065
H_85_D	85	30.1	10.23	0.92	5.908	5.482
H_120_P	120	39.2	10.02	0.25	8.037	2.045
H_120_M	120	34.8	11.52	0.26	1.094	0.081
H_120_D	120	30.2	10.98	0.32	0.643	0.046
H_145_M	145	35.0	12.05	0.24	3.369	0.718

Table S.9. General Parameters Specified Within Comsol® Model

Parameter	Comment
Hioki #1.mph *(root)*	
Global Definitions	
Parameters 1	Not really used
Default Model Inputs	Not really used
Materials	Not really used
Component 1 *(comp 1)*	
Definitions	Not really used
Geometry 1	See following table
Materials	
Copper *(mat3)*	Standard metal parameters e.g. electrical conductivity
Aluminum *(mat4)*	Standard metal parameters e.g. electrical conductivity
Au - Gold *(mat5)*	Standard metal parameters e.g. electrical conductivity
Graphite, LixC6 MCMB	Available in database; conductivity used as input variable
Electric Currents 2 *(ec2)*	
Current Conservation 1	Used as part of EC2 model
Electric Insulation 1	Insulation between all components, apart from exceptions
Initial Values 1	Not really used, as steady state model
Contact Impedance 1	Between coating and foil; used as input variable
Contact Impedance 2	Between coating and current sink (1 x 10^{-12} Ω m^2)
Contact Impedance 3	Between coating and current source (1 x 10^{-12} Ω m^2)
Ground 1	Defined as specific cylinder
Current Source 1	Defined as specific cylinder; current used as input variable
Mesh 1	
Size	Mesh element size used as input variable
Free Tetrahedral 1	Default values used
Distribution 1	Number of elements = 5, element ratio = 2
Study 1	
Step 1: Stationary	Compute in stationary mode
Solver Configurations	Not really used
Results	
Datasets	Not really used
Derived Values	Not really used
Tables	Not really used
3D Plot Group 1	Not really used
Electric Potential (ec2)	Useful images
Electric Field Norm (ec2)	Useful images
2D Plot Group 4	Not really used
Export	Data1 selected for export; potential and field norm
Reports	Not really used

Table S.10. Geometry Parameters Specified Within Comsol® Model

Component	Material	Dimensions / mm	X / mm	Y / mm	Z / mm
Geometry 1					
Foil *(blk1)*	Copper	1.6 x 1.2 x 0.010	-0.80	-0.60	-0.11
Coating *(blk2)*	MCMB	1.4 x 1.0 x 0.100	-0.70	-0.50	-0.10
Current Source *(cyl1)*	Gold	ϕ 0.020 x 0.002	-0.36	0.00	0.00
Current Drain *(cyl2)*	Gold	ϕ 0.020 x 0.002	0.36	0.00	0.00
Cylinder3 *(cyl3)*	Gold	ϕ 0.020 x 0.002	-0.24	-0.24	0.00
Cylinder 4 *(cyl4)*	Gold	ϕ 0.020 x 0.002	-0.24	-0.12	0.00
Cylinder 5 *(cyl5)*	Gold	ϕ 0.020 x 0.002	-0.24	0.00	0.00
Cylinder 6 *(cyl6)*	Gold	ϕ 0.020 x 0.002	-0.24	0.12	0.00
Cylinder 7 *(cyl7)*	Gold	ϕ 0.020 x 0.002	-0.24	0.24	0.00
Cylinder 8 *(cyl8)*	Gold	ϕ 0.020 x 0.002	-0.24	-0.24	0.00
Cylinder 9 *(cyl9)*	Gold	ϕ 0.020 x 0.002	-0.12	-0.12	0.00
Cylinder 10 *(cyl10)*	Gold	ϕ 0.020 x 0.002	0.00	0.00	0.00
Cylinder 11 *(cyl11)*	Gold	ϕ 0.020 x 0.002	0.12	0.12	0.00
Cylinder 12 *(cyl12)*	Gold	ϕ 0.020 x 0.002	0.24	0.24	0.00
Cylinder 13 *(cyl13)*	Gold	ϕ 0.020 x 0.002	0.00	-0.24	0.00
Cylinder 14 *(cyl14)*	Gold	ϕ 0.020 x 0.002	0.00	-0.12	0.00
Cylinder 15 *(cyl15)*	Gold	ϕ 0.020 x 0.002	0.00	0.00	0.00
Cylinder 16 *(cyl16)*	Gold	ϕ 0.020 x 0.002	0.00	0.12	0.00
Cylinder 17 *(cyl17)*	Gold	ϕ 0.020 x 0.002	0.00	0.24	0.00
Cylinder 18 *(cyl18)*	Gold	ϕ 0.020 x 0.002	0.12	-0.24	0.00
Cylinder 19 *(cyl19)*	Gold	ϕ 0.020 x 0.002	0.12	-0.12	0.00
Cylinder 20 *(cyl20)*	Gold	ϕ 0.020 x 0.002	0.12	0.00	0.00
Cylinder 21 *(cyl21)*	Gold	ϕ 0.020 x 0.002	0.12	0.12	0.00
Cylinder 22 *(cyl22)*	Gold	ϕ 0.020 x 0.002	0.12	0.24	0.00
Cylinder 23 *(cyl23)*	Gold	ϕ 0.020 x 0.002	0.24	-0.24	0.00
Cylinder 24 *(cyl24)*	Gold	ϕ 0.020 x 0.002	0.24	-0.12	0.00
Cylinder 25 *(cyl25)*	Gold	ϕ 0.020 x 0.002	0.24	0.00	0.00
Cylinder 26 *(cyl26)*	Gold	ϕ 0.020 x 0.002	0.24	0.12	0.00
Cylinder 27 *(cyl27)*	Gold	ϕ 0.020 x 0.002	0.24	0.24	0.00
Form Union *(fin)*					

Table S.11. Comparison Between Experimental And Model Voltage Values For Al/Cu Sample

Experiment / uV					Model / uV				
0.98	1.16	**1.33**	1.16	0.97	1.00	1.18	**1.33**	1.18	1.00
0.80	0.86	0.95	0.86	0.84	0.84	0.91	0.94	0.91	0.84
0.64	0.59	0.61	0.62	0.60	0.67	0.67	0.67	0.67	0.67
0.46	0.38	0.38	0.39	0.44	0.49	0.43	0.39	0.43	0.49
0.31	0.13	**0.00**	0.10	0.25	0.33	0.16	**0.00**	0.15	0.33

NB. Values shown in red and blue are for the voltage pins closest to the current source / sink.

Table S.12. Default Parameter Values Used In Comsol Multiphysics® Model

Parameter		Default Value
Coating conductivity	/ S m^{-1}	100
Coating thickness	/ um	100
Cu - coating Interface impedance	/ Ohm m^2	0.0001
Current source	/ mA	0.10
Coating area	/ mm^2	7.80
Foil area	/ mm^2	8.96
Mesh size		Finer

Table S.13. Mesh Element Sizes Within Comsol Multiphysics® Model

Mesh Size	Element Size / mm		Max. Element Growth Rate	Curvature Curvature	Resolution of Narrow Reg.
	Maximum	Minimum			
Normal	0.160	0.0288	1.50	0.60	0.50
Fine	0.128	0.0160	1.45	0.50	0.60
Finer	0.088	0.0064	1.40	0.40	0.70
Extra Fine	0.056	0.0024	1.35	0.30	0.85

Table S.14. Effect Of Foil Thickness And Conductivity Anisotropy On Maximum Voltage Drop (mV)

Al Foil / um	{σ_X, σ_Y, σ_Z} Diagonal Conductivities / S m^{-1}				
	{8, 8, 8}	{10, 6, 8}	{6, 10, 16}	{6, 10, 8}	{6, 10, 1}
11	18.95	23.80	11.87	14.73	54.16
119	18.95	23.65	11.93	14.80	54.30
1011	18.95	23.66	11.93	14.80	54.30

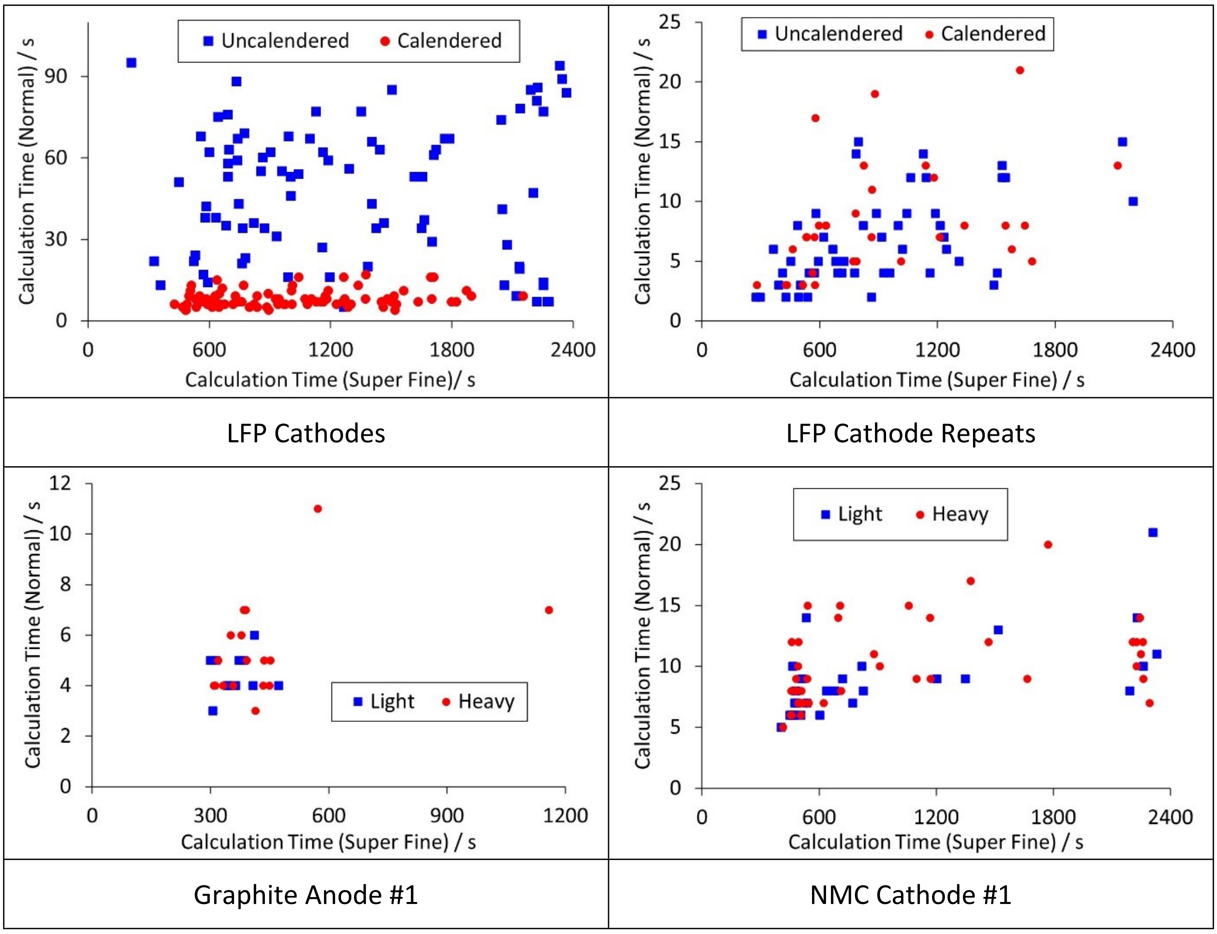

Figure S.1. Processing Times

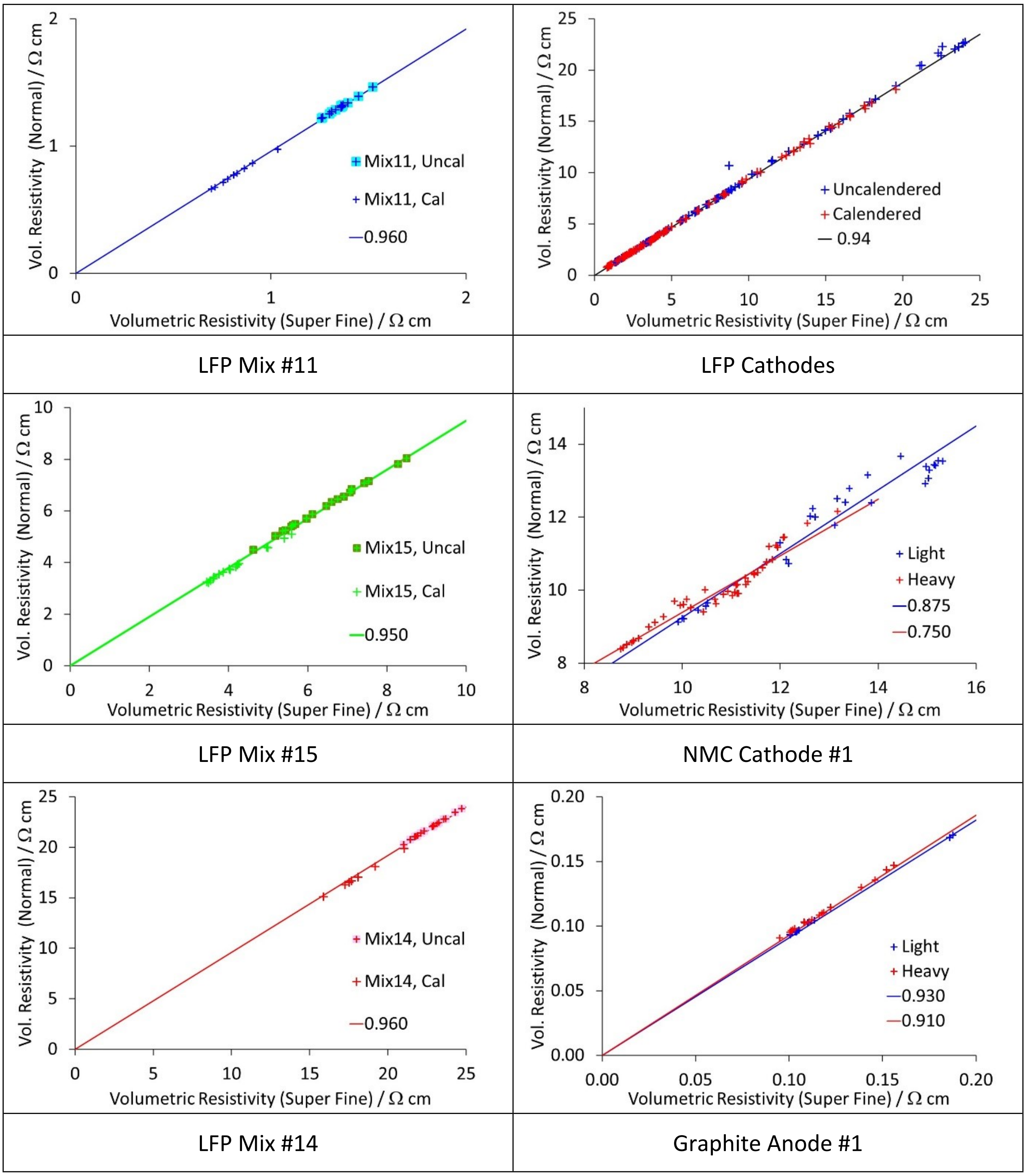

Figure S.2. Influence Of Hioki Model Parameters On Calculated Volumetric Resistivities

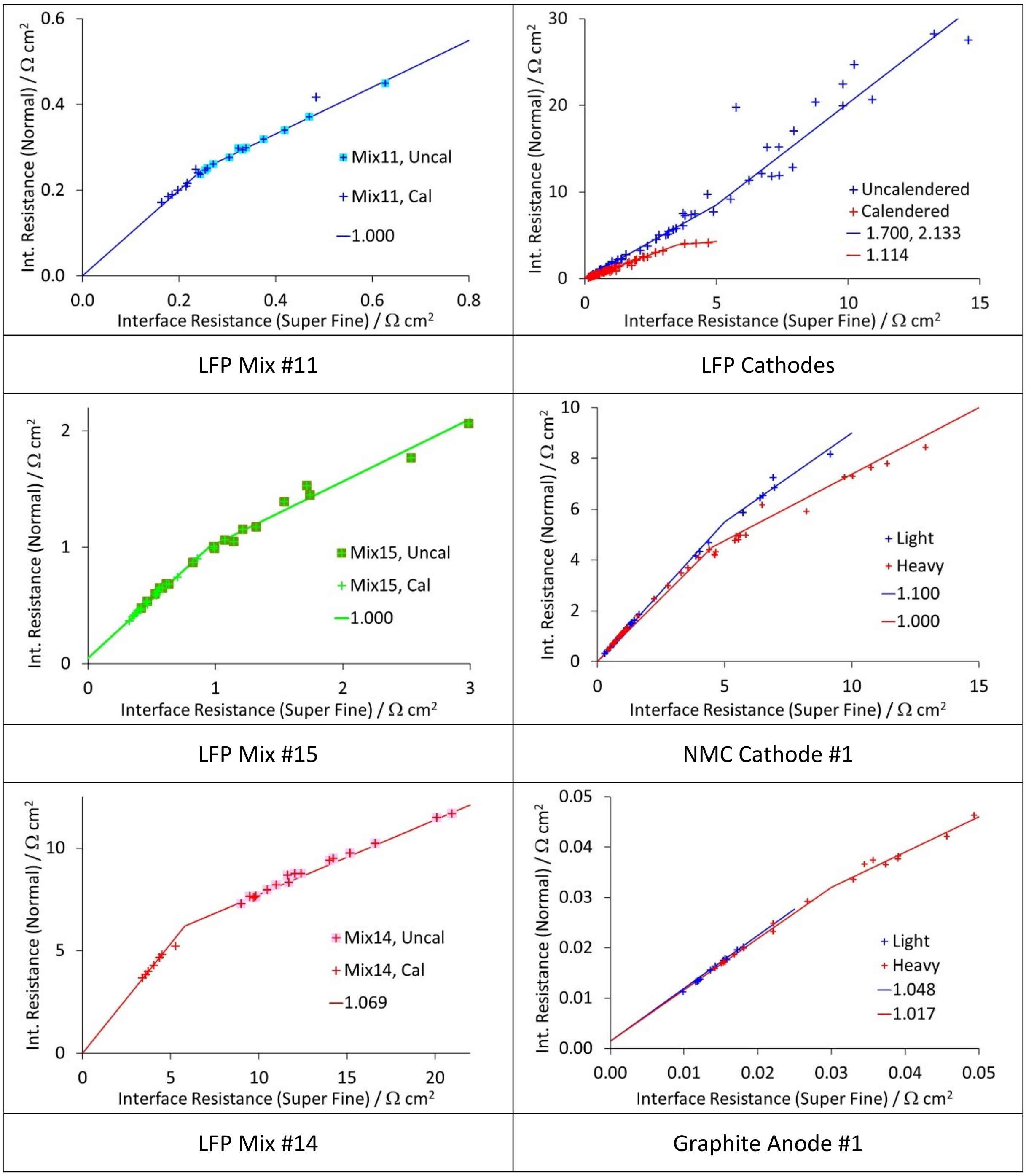

Figure S.3. Influence Of Hioki Model Parameters On Calculated Interface Resistances

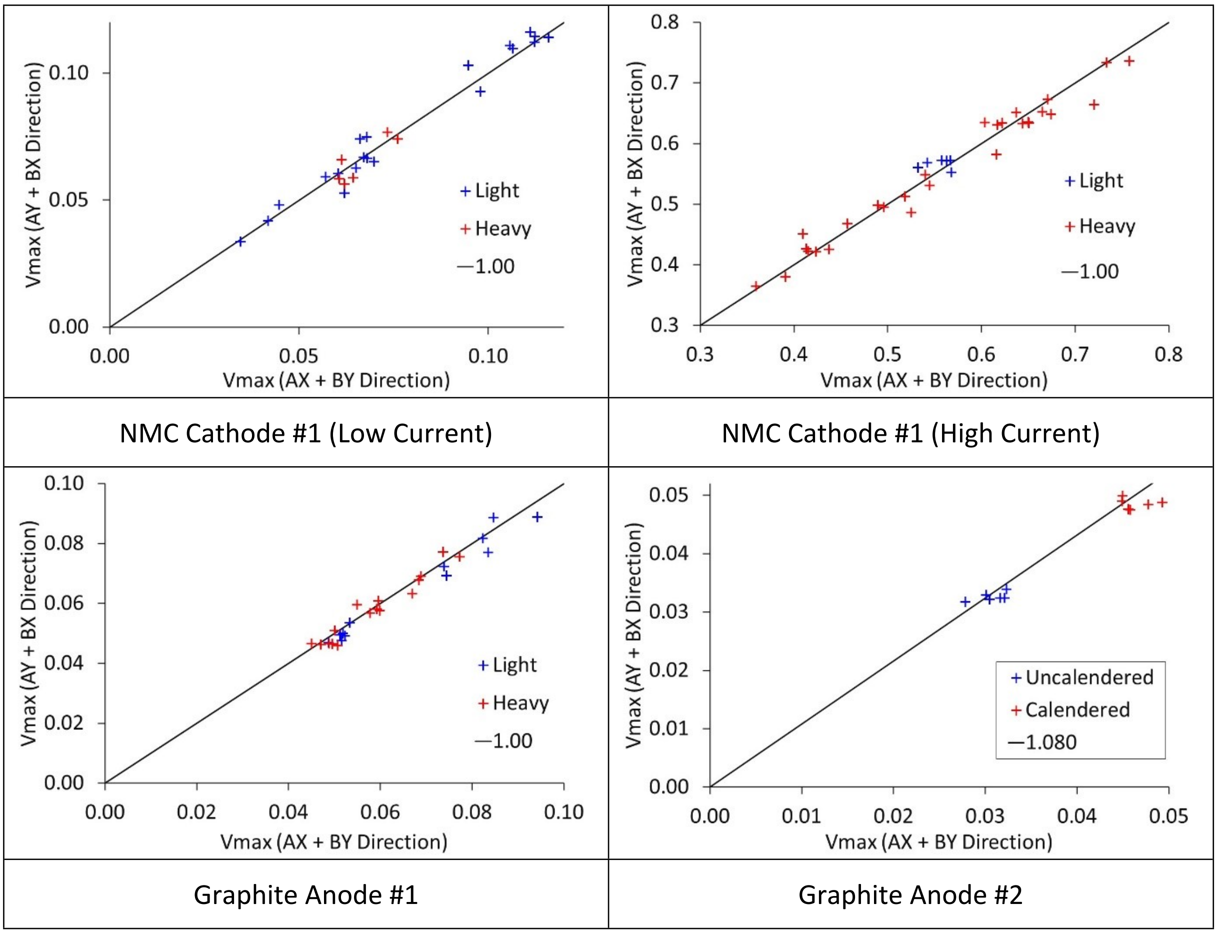

Figure S.4. Measurements Of X-Y Anisotropy Factor

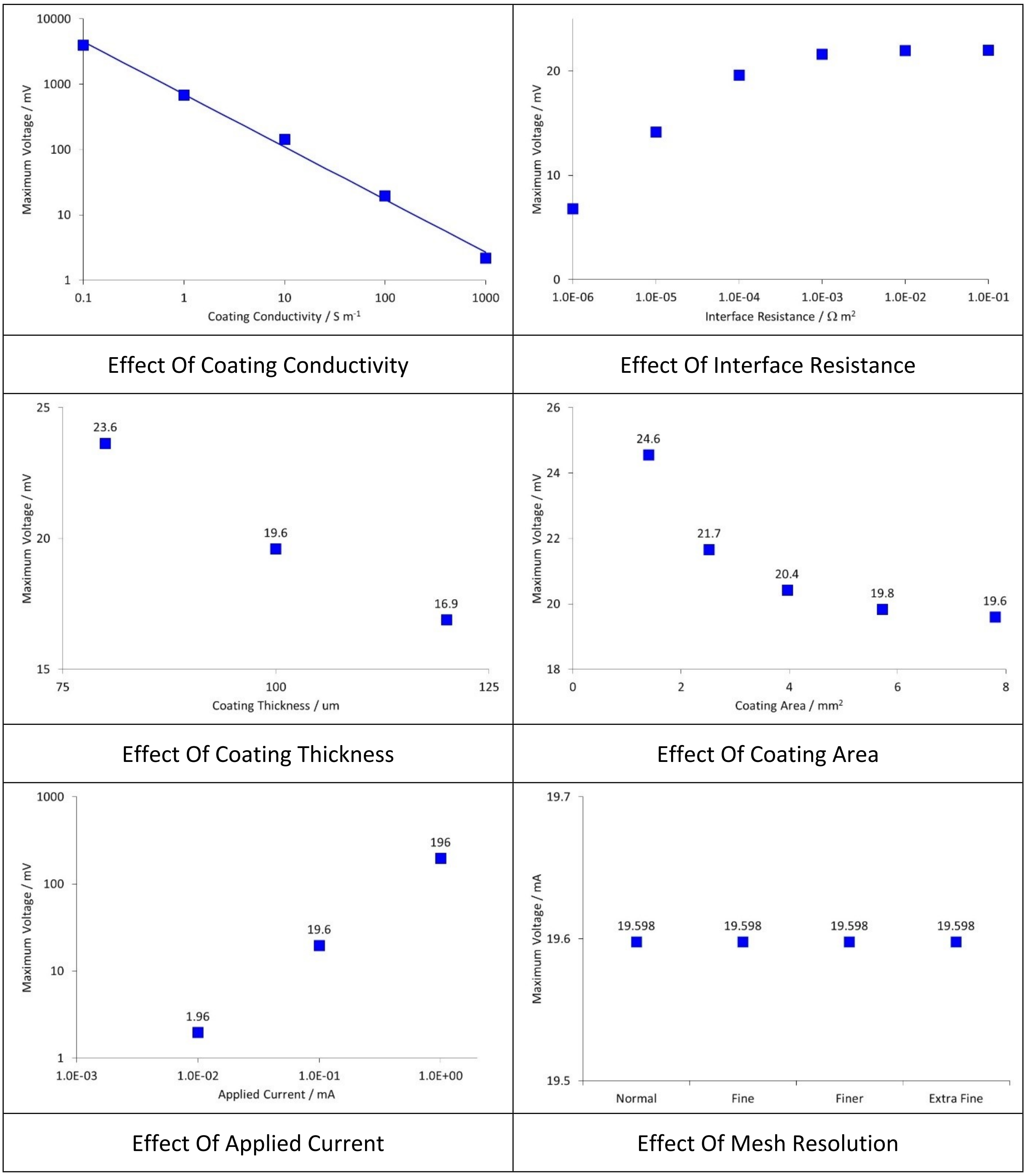

Figure S.5. Influence Of Input Parameters On Maximum Voltage Values From Comsol Model

X Direction	Z Direction
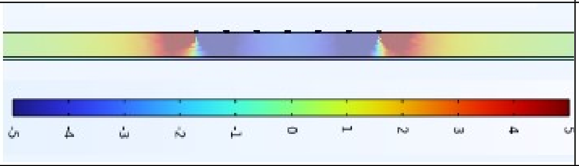	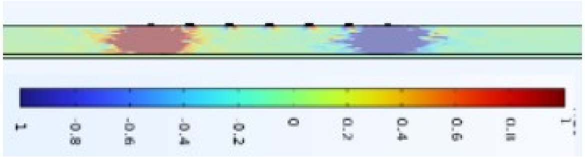
	Isotropic, Thin Foil
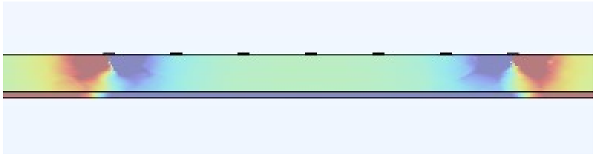	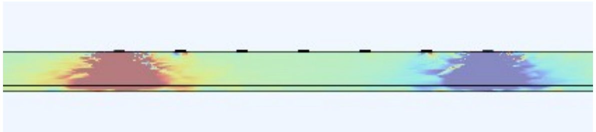
Anisotropic {6, 10, 8}, Thin Foil	Anisotropic {6, 10, 8}, Thin Foil
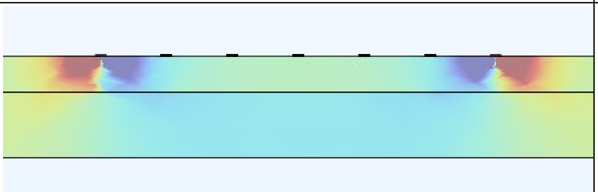	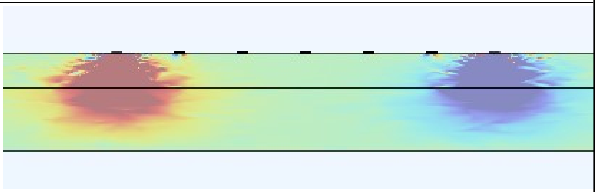
Anisotropic {6, 10, 8}, Thick Foil	Anisotropic {6, 10, 8}, Thick Foil
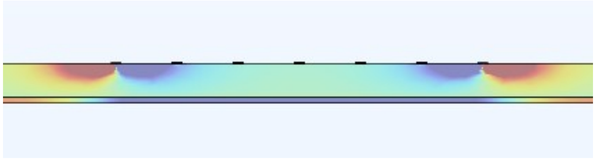	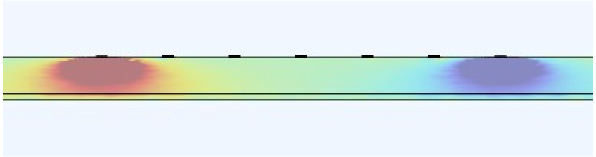
Anisotropic {6, 10, 1}, Thin Foil	Anisotropic {6, 10, 1}, Thin Foil
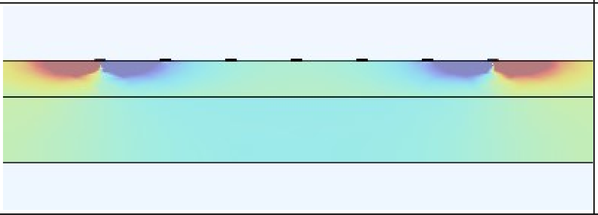	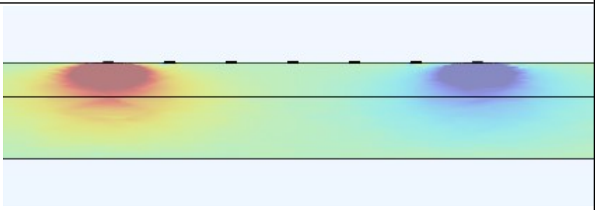
Anisotropic {6, 10, 1}, Thick Foil	Anisotropic {6, 10, 1}, Thick Foil

FigureS.6. Current Density Plots In X-Z Plane Between Current Source And Ground
